# Serum high sensitive C-reactive protein level and its correlation with lipid profile among dyspeptic patients with or without *Helicobacter pylori* infection in East Gojjam zone, Ethiopia

**DOI:** 10.3389/fcvm.2024.1470993

**Published:** 2024-09-26

**Authors:** Gelagey Baye, Bayu Wondmneh, Baye Ashenef, Mohammed Jemal, Temesgen Baylie

**Affiliations:** Department of Biomedical Science, Debre Markos University, Debre Markos, Ethiopia

**Keywords:** *Helicobacter pylori*, dyspepsia, lipid profile, high sensitive C reactive, inflammation, cardiovascular diseases

## Abstract

**Introduction:**

Dyspepsia is a group of symptoms located in the gastroduodenal area of the upper gastrointestinal tract characterized by epigastric pain, postprandial completeness, or early satiety and occasionally related to heartburn. *Helicobacter pylori* is the major causative agent of dyspepsia and gastric-related disorders; besides, it alters different metabolic processes in the human body, such as lipid metabolism and inflammatory processes. Even though dyslipidemia and inflammation are independent risk factors for cardiovascular disorders, we are assessing the interaction between serum lipids and highly sensitive C reactive protein levels among dyspeptic patients to predict potential cardiovascular disorders.

**Objectives:**

To assess serum high sensitive C reactive protein levels and its correlation with lipid profile among dyspeptic patients.

**Methods:**

A hospital-based comparative cross-sectional study was conducted from May 2022 to March 2023 in East Gojjam, Ethiopia. One hundred *Helicobacter pylori*-positive and 100 *Helicobacter pylori*-negative dyspeptic patients were included. Data were checked for completeness and entered into SPSS version 26.0 software and analyzed. The association between variables was determined by Pearson correlation analysis. A *p*-value <0.05 was considered statistically significant.

**Result:**

The mean serum high sensitive C reactive protein was 8.09 ± 7.84 mg/L, and serum high-density lipoprotein, low-density lipoprotein, total cholesterol, and triglyceride were (35.35 ± 7.5, 105.07 ± 87.63, 142.31 ± 71.31, 160.07 ± 43.06) mg/dl, respectively, for *Helicobacter pylori* positive dyspeptic patients. Among these values, high-density lipoprotein is negatively correlated with high sensitive C reactive and total cholesterol is positively correlated with high sensitive C reactive levels among *Helicobacter pylori*-infected dyspeptic patients with a *p*-value < 0.05, but in *Helicobacter pylori* negative dyspeptic patients, there is no significant correlation between lipid profile and high sensitive C reactive levels.

**Conclusion:**

Serum high sensitive C reactive levels had a negative correlation with high-density lipoprotein and a positive correlation with total cholesterol among *Helicobacter pylori*-positive dyspeptic patients. Therefore, the significant interaction between serum lipid levels and inflammation exacerbates the potential risk of cardiovascular disorders among *Helicobacter pylori*-positive dyspeptic patients.

## Introduction

Dyspepsia is a group of symptoms located in the gastroduodenal area of the upper gastrointestinal tract characterized by epigastric pain, postprandial completeness, or early satiety and occasionally related to heartburn (1).

Different factors aggravate the disease process of dyspepsia. These are *Helicobacter pylori (H. pylori)* infection, delayed gastric emptying, hypersensitivity, enteric pathogen, impaired accommodation of the stomach, and diet. Besides, lifestyle factors such as cigarette smoking, alcohol drinking, and khat chewing, and psychosocial factors like stress, anxiety, and depression (2).

*Helicobacter pylori* is the major causative agent of dyspepsia and gastric-related disorders such as peptic ulcer disease, stomach cancer, and primary stomach lymphoma. These disorders are common community health problems both in industrialized and unindustrialized countries, with greater effect in economically deprived countries due to poor hygienic circumstances. Different studies have shown that half of adults in industrialized countries are infected by *Helicobacter pylori* bacteria, but in developing countries, the severity is higher, with 90% of adults being infected by *H. pylori* (3).

Even though *H. Pylori* is the major causative agent of gastric and gastric-related disorders; it is also linked to extra-gastric complications such as cardiovascular diseases, diabetes mellitus, neurological disorders, gynecological disorders, eye diseases, dermatology, oral mucosa disorders, lung diseases, ear disorders, nose disorders, throat disorders, and blood disorders (4).

*H. Pylori* infection alters different metabolic processes in the human body. For example, it alters serum lipid levels; alterations in serum lipid levels are the greatest potential risk factors for cardiovascular disorders and metabolic syndrome (5).

Atherosclerosis, the leading cause of mortality worldwide, is the primary factor that contributes to cardiovascular disease (CVD). A well-known atherogenic lipoprotein, low-density lipoprotein, kickstarts vascular inflammation. Inflammation is an independent risk factor for manifestations of atherosclerosis and plays an important role in the underlying pathological process of atherosclerosis (6).

Different studies show the various mechanisms used by *H. pylori* bacteria to alter the serum lipid profile. Gastric colonization and attack by *H. Pylori* initiate aggravations in the lipid profile because of debilitated coagulation overflow enactment and autoimmunity because of antigenic sub-atomic mimicry between human epitopes and that of *H. pylori*, the disability of nutritional absorption, and by triggering an inflammatory reaction to the contamination (7).

*H. pylori* infection increases the circulating inflammatory markers, especially high-sensitive C-reactive protein (CRP). hs-CRP, produced in the liver and released into the blood, is a representative marker reflecting systemic inflammation. This protein is increased not only in acute but also in chronic diseases, and its slight change in chronic diseases at a low-grade subclinical level has pathophysiological relevance (5).

Various studies focused on the relationship between *H. pylori* infection and lipid profile, as well as the association between *H. pylori* infection and different inflammatory markers. However, this study aims to determine the interaction between lipid metabolism and inflammatory processes among dyspeptic patients with or without *H. pylori* infection to predict potential cardiovascular risk, even though dyslipidemia and inflammation are well-known independent risk factors for the onset of cardiovascular disease (CVD).

Numerous complex biological pathways are involved in the relationship between lipid metabolism and inflammation. Initially, significant pro-inflammatory cytokines, like TNF-α and interleukin 6 (IL-6), are crucial for controlling lipid metabolism (4, 8). Tumor-necrotizing factor alpha (TNF-α) can increase the amount of free fatty acids in the bloodstream by impairing lipoprotein lipase's function and aggravating adipocytes' fat breakdown process. The pro-inflammatory cytokine IL-6 modifies lipoprotein function and raises hepatic triglyceride synthesis (9, 10). However, inflammation suppresses nuclear receptors like peroxisome proliferator-activated receptor (PPAR), which is important for controlling lipid metabolism and hence upsets lipid homeostasis. When inflammatory signals activate the transcription factor nuclear factor kappa (NF-*κ*B), they down regulate genes related to fat metabolism while enhancing the expression of pro-inflammatory genes (11). Furthermore, eicosanoids, lipid mediators sourced from arachidonic acid, have the ability to affect insulin sensitivity and adipocyte activity, thus establishing a connection between lipid metabolism and inflammation (10).

Besides, lipoproteins have the ability to control inflammation and immune cell activity. Due to its ability to transport bioactive lipids, anti-inflammatory and antioxidant proteins, and regulate immune cell activation by mediating cellular cholesterol efflux and reorganizing pattern recognition receptor (PRR) and related co-receptors in membrane lipid rafts, high-density lipoproteins (HDL) in particular are known to possess a variety of immune-modulatory and anti-inflammatory properties (10, 12).

The pro-oxidative enzyme myeloperoxidase (MPO) and its subsequent formation of malondialdehyde (MDA) can be inhibited by paraoxonase 1 (PON1), an HDL-associated antioxidant enzyme known to neutralize oxidized phosphatidylcholine moieties and prevent the accumulation of lipid hydroperoxides in lipoproteins. MDA has the ability to covalently crosslink HDL-associated apolipoprotein A1 (apoA1) and impede the positive effects of HDL on inflammation and cholesterol-effluxing properties (11).

Low-grade inflammation is characterized by a slight chronic elevation of inflammatory markers in the blood, but not to the same degree as acute inflammation. High-sensitivity C-reactive protein (hs-CRP), known as a classic indicator for low-grade inflammation, has undergone extensive research among diverse groups of inflammatory biomarkers and has drawn the greatest attention because of its potential as a reliable and affordable predictor for CVD (13–17).

## Materials and methods

A hospital-based cross-sectional study was conducted at Dejen Primary Hospital, Debre Markos Referral Hospital, and Lumamie Primary Hospital from May 2022 to March 2023, East Gojjam, Ethiopia. In this study, 100 *H. pylori*-positive and 100 *H. pylori*-negative dyspeptic patients were selected during a health check-up in the outpatient department (OPD) by the purposive sampling technique as those who fulfilled inclusion criteria. All dyspeptic patients attending the three hospitals during the data collection period were included in the study, but subjects with liver disease, renal dysfunction, obesity, diabetes, cardiovascular disorders, malignancy, rheumatoid arthritis, and gouty arthritis who received the antihyperlipidemic drug and patients with a history of *H. Pylori* eradication therapy were excluded from the study. After we obtained informed consent from the study participants, 5 ml of blood was drawn, and the blood was allowed to stand for 30 min for complete clotting, centrifuged at 3,000 rpm for 15 min to extract serum, and stored at −70°C before the day of analysis.

### Laboratory tests

Using a chromatographic immunoassay for the qualitative detection of *H. Pylori* antigen in the human fecal material [Joaquim Costa 18 2a planta. 08390 Montgat (Barcelona) SPAIN], *H. Pylori* was found using a linear *H. Pylori* Ag cassette. The Friedwald formula was used to determine the serum levels of low-density lipoprotein (LDL), while the Cobas C 501 chemical analyzer (Roche Diagnostic, USA) was used to assess the levels of total cholesterol, high-density lipoprotein (HDL), triglycerides (TG), and high sensitive C reactive protein (hs-CRP). At the Ethiopian Public Health Institute in Addis Ababa, Ethiopia, all biochemical analyses were completed. HDL (40–60 mg/dl), TG (<130 mg/dl), TC (<200 mg/dl), LDL (<130 mg/dl), and hs-CRP (<5 mg/L) were the reference ranges for adults.

### Data processing and analysis

Version 26.0 of the SPSS software suite was used to analyze the data. The socio-demographic information is presented using basic descriptive statistics. However, the relationship between the variables was described using Pearson chi-square and Pearson correlation. The mean ± standard deviation is used to represent continuous variables having a normal distribution. To compare the variables, an independent student *t*-test was employed. A 95% confidence level *p*-value of less than 0.05 designates a statistically significant result.

## Result

Socio-demographic data of dyspeptic patients: This study was conducted on 100 *H. pylori*-positive and 100 *H. pylori*- negative dyspeptic patients. The average age of *H. pylori*-positive dyspeptic patients was 41.59 ± 12.4 and 35.54 ± 12.59. Of the total dyspeptic patients included in this study, 76 participants were female and the remaining 126 participants were male. The majority of dyspeptic patients were alcohol users 74 participants in *H. pylori* positive and 71 participants in *H. pylori*-negative dyspeptic patients. The socio-demographic variables are summarized in Table 1.

**Table 1:** Socio-demographic data of dyspeptic patients.

Variables	Category of variable	*H. Pylori* positive (*n* = 100)		*H. pylori* negative (*n* = 100)		*P*-value
		Frequency	%	Frequency	%	
Sex	Males	58	58	66	66	0.24
	Females	42	42	34	34	
Educational status	Illiterate	28	28	19	19	0.32
	Primary education	32	32	42	42	
	Secondary education	25	25	27	27	
	College/university	15	15	12	12	
Residency	Urban	44	44	38	38	0.38
	Rural	56	56	62	62	
Income	Low	23	23	31	31	0.37
	Medium	60	60	51	51	
	High	17	17	18	18	
Age		41.59 ± 12.4	50	36.12 ± 12.8	50	0.24
Alcohol use	Yes	74	71	71	74	0.38
	No	26	29	29	26	
Smoking	Yes	14	10	10	14	0.64
	No	86	90	90	86	
Khat chewing	Yes	22	22	13	13	0.09
	No	78	78	87	87	

All categorical variables are determined by number and percent. The average age of the study participant is presented as mean ± SD. *H. pylori*, *Helicobacter pylori*.

The average serum hs-CRP levels and lipid profile among dyspeptic patients with and without *H. pylori* infection:

The average serum hs-CRP levels of *H. pylori* positive and negative dyspeptic patients were 8.09 ± 7.84 mg/L and 2.71 ± 2.51 mg/L respectively with a standard range of hs-CRP (<5 mg/L) in adults. The average serum HDL levels of *H. pylori* positive and negative dyspeptic patients were 35.35 ± 7.5 mg/dl and 44.64 ± 6.69 mg/dl respectively with a standard range of HDL (40–60 mg/dl) in adults.

An independent *t*-test was also conducted to compare the mean serum hs-CRP levels and lipid profiles (TG, TC, HDL, and LDL) between *H. pylori*-positive and negative dyspeptic patients. All serum lipid profiles and hs-CRP levels were significantly different between *H. pylori*-positive and *H. pylori*-negative dyspeptic patients with *P*-value <0.05. The summary is presented in Table 2.

**Table 2:** The comparison of serum lipid profile and hs-CRP between *H. pylori*-positive and *H. pylori*-negative dyspeptic patients.

Variables	*H. pylori*-positive (*n* = 100)	*H. pylori*-negative (*n* = 100)	*p*-value
HDL mg/dl	35.35 ± 7.5	44.64 ± 6.69	0.000
LDL mg/dl	105.07 ± 87.63	86.43 ± 26.64	0.043
TG mg/dl	142.31 ± 71.31	113.05 ± 90.6	0.012
TC mg/dl	160.07 ± 43.06	148.39 ± 34.53	0.036
hs-CRP mg/L	8.09 ± 7.84	2.71 ± 2.51	0.000

*H. pylori*, *Helicobacter pylori*. Serum hs-CRP and lipid profiles are presented as mean ± SD. HDL, high-density lipoprotein; TC, total cholesterol; LDL, low-density lipoprotein; TG, triglyceride; hs-CRP, highly sensitive c-reactive protein.

**Figure 1:**
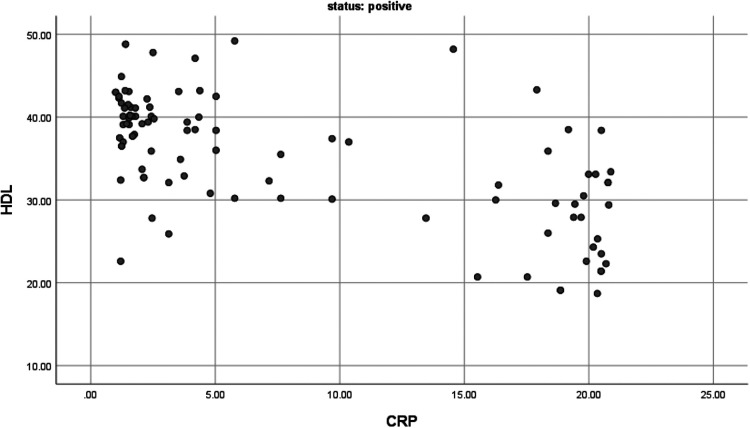
The correlation between HDL and CRP among *H. Pylori* positive dyspeptic patients (*r* = −0.64 and *P*-value <0.01 with *n* = 100).

**Figure 2:**
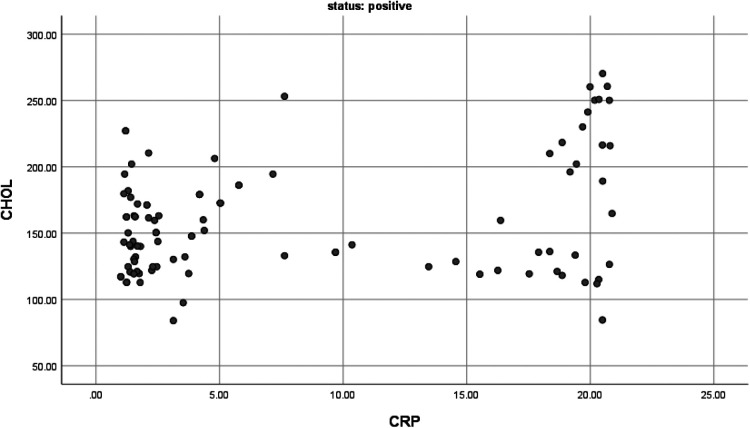
The correlation between CHOL and CRP among *H. Pylori* positive dyspeptic patients (*r* = 0.29 and *P*-value <0.01 with *n* = 100).

The correlation between serum hs-CRP levels and lipid profile among dyspeptic patients: Pearson correlation analysis was conducted to evaluate the association between hs-CRP and lipid profile (TG, HDL, LDL, and TC) among dyspeptic patients with and without *H. pylori* infection. In *H. pylori*-positive dyspeptic patients, serum TC levels were found positive, moderate in strength, and a statistically significant relationship with serum hs-CRP levels (*r* = 0.293, *p* < 0.05), and serum HDL levels were also found statistically significant but had a negative relationship with serum hs-CRP levels (*r* = −0.64, *p* < 0.01) (Figures 1, 2). This finding suggests that the higher hs-CRP levels in *H. pylori*-infected dyspeptic patients are more likely to have higher TC and lower HDL levels. However, in *H. pylori*-negative dyspeptic patients’ hs-CRP levels have an insignificant correlation with lipid profiles with *P*-value >0.05. The correlation between serum hs-CRP levels and lipid profile among dyspeptic patients is summarized in Table 3.

**Table 3:** The correlation between serum hs-CRP levels and lipid profile among *H. pylori*-positive dyspeptic patients.

Lipid profile	Pearson correlation	hs-CRP
HDL	*r*	−0.64
	*P*-value	0.000*
LDL	*r*	0.9
	*P*-value	0.37
TG	*r*	0.194
	*P*-value	0.053
TC	*r*	0.293
	*P*-value	0.003*

HDL, high-density lipoprotein; TC, total cholesterol; LDL, low-density lipoprotein; TG, triglyceride; hs-CRP, highly sensitive c-reactive protein.

* Correlation is significant at *p*-value <0.05 level (2-tailed). Serum hs-CRP and lipid profiles are presented as mean ± SD.

## Discussion

In our study, the average age of *H. pylori*-positive dyspeptic patients was 41.56 years, which was higher than the average age of *H. pylori*-negative dyspeptic patients (36.1 years). This was in line with a study conducted in Indonesia (18) and Jimma (19). The possible explanation for this age-related correlation between *H. pylori* infection and susceptibility to infectious diseases is the weakened immune system that accompanies aging. One reason the immune system is unable to effectively defend the body against a foreign body is that aging causes several immune system components to become dysregulated. In addition, a longer life expectancy raises the risk of developing an *H. pylori* infection because of increased exposure to the germs (20).

The majority of dyspeptic participants in our study—both those with and without *H. pylori* infection—were alcohol consumers. A study that was comparable to this one was published in Similar conclusions were drawn from research conducted in West Africa (21), and Hosanna (22). This could be because drinking alcohol increases the risk of developing an *H. pylori* infection and increases the prevalence of dyspepsia in a number of ways. First, alcohol causes inflammation by weakening the gastric mucosal membrane and increasing the mucosa's permeability. Interleukin-8 (IL-8), a cytokine released by neutrophils and macrophages during inflammation, binds with its receptor to promote further inflammation. Interleukin-8 is linked to HP0638, an external inflammatory protein virulence factor that makes *H. pylori* more adherence-prone and so promotes bacterial colonization (23).

The serum lipid profile (HDL, LDL, TC, and TG) was a statically difference between *H. pylori* positive and negative dyspeptic patients. In *H. pylori*-positive dyspeptic patients, the serum levels of LDL, TC, and TG were higher than *in H. pylori*-negative dyspeptic patients but for HDL the value is vice versa. A similar study was reported in the studies conducted in Jimma, Iraq, and China (19, 24, 25). This may result from changes in lipid metabolism after infection with *H. pylori,* which produces numerous virulence factors, including cytotoxic-associated genes (CagA) and vacuolating cytotoxic A (VacA). These virulence factors generate host factors such as interleukins, interferon-gamma, tumor necrosis factor, T and B lymphocytes, and phagocytic cells, as well as inflammation and cellular damage to host cells (26). In particular, TNF-α inhibits lipoprotein lipase, which causes lipids from tissues to be mobilized into the blood and raises serum levels of TG, TC, and LDL (11). Additionally, the lipid and protein composition of HDL are significantly altered by inflammation brought on by an H. pylori infection, which lowers the concentration of HDL (27).

In our investigation, we found that in *H. pylori*-positive dyspeptic patients, there was a substantial link between the lipid profile, notably HDL and TC, and the inflammatory markers, specifically hs-CRP levels. Analogous research and reviews demonstrating this correlation have been published in different countries (9, 11, 28, 29). This could be because a variety of lipid species, such as sterols, complex lipids, lipoproteins, fatty acids, and their metabolites have been shown to have immunomodulatory and pro- and anti-inflammatory qualities (12).

Due to its ability to transport bioactive lipids, anti-inflammatory and anti-oxidant proteins, and regulate immune cell activation by mediating cellular cholesterol efflux and reorganizing pathogen recognition receptors (PRRs) and related coreceptors in membranes lipid rafts, higher-density lipoproteins (HDL) in particular are known to possess a variety of immunomodulatory and anti-inflammatory properties (30).

Various research works have documented the significance of HDL in maintaining anti-inflammatory and cholesterol-accepting capacities. They discovered that pro-oxidative enzyme myeloperoxidase (MPO) can be inhibited by paraoxonase 1 (PON1), an HDL-associated antioxidant enzyme known to neutralize oxidized phosphatidylcholine moieties and prevent the accumulation of lipid hydroperoxides in lipoproteins. Besides, HDL inhibits the formation of malondialdehyde (MDA), which can covalently crosslink HDL-associated apolipoprotein A1 (apoA1) and impair the positive anti-inflammatory and cholesterol-effluxing properties of HDL (12, 31).

## Conclusion

Different studies focus on the effect of *H. pylori* infection on lipid profile and inflammatory markers rather than the interaction between lipid profile and inflammation among dyspeptic patients (5, 26, 32, 33). However, our study focuses on the correlation between lipid profiles and inflammatory markers among dyspeptic patients. Based on our study result we can conclude that dyslipidemia and inflammation interact with each other, so provoking the potential risk of cardiovascular disorders.

## Data Availability

The raw data supporting the conclusions of this article will be made available by the authors, without undue reservation.
